# *Cryptosporidium*: from Molecules to Disease

**DOI:** 10.3201/eid1009.040566

**Published:** 2004-09

**Authors:** Timothy J. Wade

**Affiliations:** * U.S. Environmental Protection Agency, Chapel Hill, North Carolina, USA

**Keywords:** *Cryptosporidium*: From Molecules to Disease, R.C. Andrew Thomson, Anthony Armson,Una M. Ryan

The protozoan parasite, *Cryptosporidium*, has recently emerged as a human pathogen. It was unidentified or unrecognized as a cause of illness in humans until 1976. Since then, it has caused gastrointestinal illness around the world. Its small size, low infectious dose, resistance to chlorination, and durability in the environment has made it a uniquely challenging organism for environmental scientists and public health professionals.

This book includes full text of abstracts and invited papers from an international conference held in Australia in October 2001. More than 100 scientists from more than 15 countries contributed to the conference.

The "from molecules" aspect of the book, which addresses molecular and biochemical features of the life cycle, infection, and detection of *Cryptosporidium*, gives a complete picture with detailed papers and abstracts of subjects, including pathogenesis and immune response, cell culture methods, detection methods, and molecular taxonomy. The main focus of the book is on descriptions and evaluations of traditional and novel methods to detect and differentiate *Cryptosporidium*. Papers are also included that describe methods of detecting *Cryptosporidium* in environmental water samples, detail surveys that determine the occurrence of *Cryptosporidium* in water supplies, and explain how to acquire laboratory accreditation for testing water samples.

The book focuses less on understanding the public health aspects of *Cryptosporidium*, its epidemiology, and treatment for the illness it causes. Notably absent are descriptions of serologic assays used for detecting *Cryptosporidium* in surveillance and epidemiologic studies. Recent studies have identified a high seroprevalence in the general population, which indicates that infection may be widespread (*1**–**5*). Including examples of quantitative microbial risk assessments would have been useful (*6*). These assessments are logical extensions of the valuable human infectivity studies described in several papers in the book. The treatment portion presents interesting results of randomized trials of nitroaxanide therapy but is otherwise limited.

The organization and grouping of the papers and abstracts were confusing. An introduction and summary for each section to help the reader identify and assimilate the information in an organized manner would have been helpful.

Despite these shortcomings, this book assembles and summarizes an impressive array of recent advances in *Cryptosporidium* research. I recommend this book for laboratory scientists, microbiologists, laboratory technicians, and water-quality professionals. Medical professionals involved with research to detect and differentiate *Cryptosporidium* will likely find this book useful. Because of the technical nature of the papers and the emphasis on microbiologic methods, the book will be less useful for public health professionals, risk managers, and epidemiologists. Because of the rapid progress of *Cryptosporidium* research, I recommend using this book as one reference but also conducting a broad search of current literature for new studies or additional advances.
